# Health of Special Immigrant Visa holders from Iraq and Afghanistan after arrival into the United States using Domestic Medical Examination data, 2014–2016: A cross-sectional analysis

**DOI:** 10.1371/journal.pmed.1003083

**Published:** 2020-03-31

**Authors:** Gayathri S. Kumar, Clelia Pezzi, Simone Wien, Blain Mamo, Kevin Scott, Colleen Payton, Kailey Urban, Stephen Hughes, Lori Kennedy, Nuny Cabanting, Jessica Montour, Melissa Titus, Jenny Aguirre, Breanna Kawasaki, Rebecca Ford, Emily S. Jentes

**Affiliations:** 1 Centers for Disease Control and Prevention, Division of Global Migration and Quarantine, Immigrant, Refugee, and Migrant Health Branch, Atlanta, Georgia, United States of America; 2 Minnesota Department of Health, Saint Paul, Minnesota, United States of America; 3 Thomas Jefferson University, Philadelphia, Pennsylvania, United States of America; 4 Bureau of Tuberculosis Control, New York State Department of Health, Albany, New York, United States of America; 5 Colorado Department of Public Health and Environment, Disease Control and Environmental Epidemiology Division, Refugee Health Program, Denver, Colorado, United States of America; 6 Office of Refugee Health, Center for Infectious Diseases, California Department of Public Health, Sacramento, California, United States of America; 7 Texas Department of State Health Services, Austin, Texas, United States of America; 8 Marion County Public Health Department, Indianapolis, Indiana, United States of America; 9 Illinois Department of Public Health, Refugee Health Program, Chicago, Illinois, United States of America; 10 University of Louisville Division of Infectious Diseases, Louisville, Kentucky, United States of America; International Organization for Migration, SRI LANKA

## Abstract

**Background:**

Since 2008, the United States has issued between 2,000 and 19,000 Special Immigrant Visas (SIV) annually, with the majority issued to applicants from Iraq and Afghanistan. SIV holders (SIVH) are applicants who were employed by, or on behalf of, the US government or the US military. There is limited information about health conditions in SIV populations to help guide US clinicians caring for SIVH. Thus, we sought to describe health characteristics of recently arrived SIVH from Iraq and Afghanistan who were seen for domestic medical examinations.

**Methods and findings:**

This cross-sectional analysis included data from Iraqi and Afghan SIVH who received a domestic medical examination from January 2014 to December 2016. Data were gathered from state refugee health programs in seven states (California, Colorado, Illinois, Kentucky, Minnesota, New York, and Texas), one county, and one academic medical center and included 6,124 adults and 4,814 children. Data were collected for communicable diseases commonly screened for during the exam, including tuberculosis (TB), hepatitis B, hepatitis C, malaria, strongyloidiasis, schistosomiasis, other intestinal parasites, syphilis, gonorrhea, chlamydia, and human immunodeficiency virus, as well as elevated blood lead levels (EBLL). We investigated the frequency and proportion of diseases and whether there were any differences in selected disease prevalence in SIVH from Iraq compared to SIVH from Afghanistan. A majority of SIV adults were male (Iraqi 54.0%, Afghan 58.6%) and aged 18–44 (Iraqi 86.0%, Afghan 97.7%). More SIV children were male (Iraqi 56.2%, Afghan 52.2%) and aged 6–17 (Iraqi 50.2%, Afghan 40.7%). The average age of adults was 29.7 years, and the average age for children was 5.6 years. Among SIV adults, 14.4% were diagnosed with latent tuberculosis infection (LTBI), 63.5% were susceptible to hepatitis B virus (HBV) infection, and 31.0% had at least one intestinal parasite. Afghan adults were more likely to have LTBI (prevalence ratio [PR]: 2.0; 95% confidence interval [CI] 1.5–2.7) and to be infected with HBV (PR: 4.6; 95% CI 3.6–6.0) than Iraqi adults. Among SIV children, 26.7% were susceptible to HBV infection, 22.1% had at least one intestinal parasite, and 50.1% had EBLL (≥5 mcg/dL). Afghan children were more likely to have a pathogenic intestinal parasite (PR: 2.7; 95% CI 2.4–3.2) and EBLL (PR: 2.0; 95% CI 1.5–2.5) than Iraqi children. Limitations of the analysis included lack of uniform health screening data collection across all nine sites and possible misclassification by clinicians of Iraqi and Afghan SIVH as Iraqi and Afghan refugees, respectively.

**Conclusion:**

In this analysis, we observed that 14% of SIV adults had LTBI, 27% of SIVH had at least one intestinal parasite, and about half of SIV children had EBLL. Most adults were susceptible to HBV. In general, prevalence of infection was higher for most conditions among Afghan SIVH compared to Iraqi SIVH. The Centers for Disease Control and Prevention (CDC) *Guidelines for the US Domestic Medical Examination for Newly Arriving Refugees* can assist state public health departments and clinicians in the care of SIVH during the domestic medical examination. Future analyses can explore other aspects of health among resettled SIV populations, including noncommunicable diseases and vaccination coverage.

## Abstract 2

In a cross-sectional analysis, Gayathri S. Kumar and colleagues assess the health of Special Immigrant Visa holders from Iraq and Afghanistan after arrival into the United States using domestic medical examination data (2014–2016).

## Author summary

**Why was this study done?:**

- Since 2008, the US has typically issued between 2,000 and 19,000 Special Immigrant Visas (SIV) annually, the majority to applicants from Iraq and Afghanistan.
- SIV holders (SIVH) are eligible for many of the same benefits as refugees under the US Refugee Admissions Program, including a domestic medical examination within 90 days of US arrival.
- Information about the health of SIV populations is limited and would be beneficial for US clinicians who see SIVH in their clinics.

**What did the researchers do and find?:**

- In this cross-sectional analysis, data were gathered from domestic medical examinations of 10,938 SIVH from nine sites for diseases commonly screened for during the exam.
- We observed that 14.4% of SIV adults had LTBI, 26.9% of SIVH had at least one intestinal parasite, and about half of SIV children had elevated blood lead levels (EBLL). Most adults were susceptible to hepatitis B virus (HBV).
- In general, prevalence of infection was higher for most conditions among Afghan SIVH compared to Iraqi SIVH.

**What do these findings mean?:**

- CDC’s *Guidelines for the US Domestic Medical Examination for Newly Arriving Refugees* can assist state public health departments and clinicians in the care of SIVH during the routine domestic medical examination.

- Clinicians can ensure that SIVH receive appropriate screening and follow-up, paying particular attention to Afghan SIVH given their increased risk of several communicable diseases and EBLL (among children).
- Limitations of the analysis included lack of uniform health screening data collection across all nine sites and possible misclassification by clinicians of Iraqi and Afghan SIVH as Iraqi and Afghan refugees, respectively.

## Introduction

Since 2008, the US has issued between 2,000 and 19,000 Special Immigrant Visas (SIV) annually, the majority to applicants from Iraq and Afghanistan [1]. SIV holders (SIVH) are applicants who were employed by, or on behalf of, the US government or the US military—notably as translators and interpreters with the US Armed Forces—and can include their immediate families [2]. Like refugees and other immigrants, all SIVH are required to receive a medical screening exam overseas according to the Technical Instructions written by the Centers for Disease Control and Prevention (CDC) [3]. SIVH are eligible for many of the same benefits as refugees under the US Refugee Admissions Program (USRAP); these benefits may include resettlement assistance and health-related benefits after arrival in the US [4,5]. SIVH have the option to receive these benefits before or after arrival. In addition, the CDC also recommends that SIVH and refugees receive a domestic medical examination within 90 days after arrival to the US [6]. However, SIVH do not have access to the overseas health interventions available to refugees, such as vaccines or parasite treatments, before departure to the US. Furthermore, SIVH must meet the requirements for vaccinations in CDC’s Vaccination Technical Instructions for immigrants, whereas refugees may be granted a waiver for vaccinations [7].

Almost 80,000 SIVH received USRAP benefits from 2008 to 2018, including close to 18,500 from Iraq and 58,000 from Afghanistan [1]. There is little information about health conditions in SIV populations despite the high number who have elected USRAP benefits. Multiple studies have reported on the health of Iraqi and Afghan refugee populations [8–11]. However, SIVH encounter different circumstances while living abroad compared to refugees. SIVH are more likely to live in urban settings as opposed to camps before immigration and therefore likely to encounter different risks of disease. SIVH have faced threats to themselves and their families from militant groups while serving in roles such as interpreters or translators for the US government or military and often have gone into hiding [12, 13]. Therefore, it is possible that SIVH had less access to clinical and preventive health services, despite having the financial capacity to pay for these services.

Clinicians should follow CDC’s *Guidelines for the US Domestic Medical Examination for Newly Arrived Refugees* during the domestic medical examination for SIVH [14]. They should (1) review SIVH’s Department of State (DS) overseas medical examination forms, (2) obtain a complete medical history, (3) conduct a physical examination, (4) screen for communicable health conditions, such as tuberculosis (TB), (5) identify other conditions that may adversely affect resettlement or require further care, such as noncommunicable diseases (including mental health concerns), (6) provide preventive health interventions, such as immunizations, and (7) link the SIVH with appropriate care and follow-up [14]. The CDC guidelines are informed by disease epidemiology of the country of birth and country of residence prior to US resettlement. However, each state or community can adapt the screening guidelines to suit local circumstances and resources. Increasing clinician knowledge about common health conditions encountered in SIVH, including differences between Iraqi and Afghan SIVH, may facilitate diagnostic screening, physical examination, and referrals to additional healthcare providers in the US. To the authors’ knowledge, this information is not currently available. Thus, we sought to describe health characteristics of recently arrived SIVH from Iraq and Afghanistan who were seen for domestic medical examinations. Specifically, we investigated the frequency and proportion of diseases commonly screened for in the domestic medical examination and, for certain diseases, whether there were any differences in prevalence for SIVH from Iraq compared to SIVH from Afghanistan.

## Methods

### Analysis population and data collection

This cross-sectional analysis included data from Iraqi and Afghan SIVH who received a domestic medical examination between January 2014 and December 2016. CDC collaborated with nine partners as part of a cooperative agreement (CK12-1205) to collect domestic medical examination data for analysis. Partners included the state refugee health programs in California, Colorado, Illinois, Kentucky, Minnesota, New York, and Texas; local partners in Marion County, Indiana; and an academic health center in Philadelphia, Pennsylvania. Data were obtained from partner refugee health databases. Some partners obtained individual-specific data from external programs (e.g., TB or lead prevention) in their jurisdictions. As part of a larger data collection effort, this analysis was not guided by a specific prospective analysis plan. However, the variables collected for analysis were outlined in a protocol used for ethical determination and shared with site partners contributing data. After data collection, we assessed the quality and availability of our data and identified analyses appropriate for our data, including this analysis describing health of SIVH. This analysis was determined to be non-research surveillance by a CDC human subjects advisor, and therefore formal Institutional Review Board (IRB) review was not required. This analysis is reported as per the Strengthening the Reporting of Observational Studies in Epidemiology (STROBE) guideline (S1 STROBE Checklist).

Demographic information (sex, age, and primary language spoken by the SIVH or used by the interpreter during the exam), nationality, and country of last residence were either provided by the site or, if a unique identifier was provided in the data set, obtained from matched records in CDC’s Electronic Disease Notification (EDN) system. EDN is a centralized reporting system that notifies US state and local health departments and screening clinics of the arrival of immigrants with health conditions requiring medical follow-up, including TB-related conditions, and all refugees. Sites also reported timing of the domestic medical examination relative to US arrival and whether a particular health condition was screened for. Screening test results were collected for TB, hepatitis B, hepatitis C, malaria, strongyloidiasis, schistosomiasis, presence of other intestinal parasites, syphilis, gonorrhea, chlamydia, HIV, and elevated blood lead levels (EBLL). Data were collected for these conditions, as they are recommended for screening according to CDC’s *Guidelines for the US Domestic Medical Examination for Newly Arriving Refugees* [14]. For most conditions, we were unable to collect detailed information on the method of screening used by partners. We assumed the sites conducted screening or testing according to the CDC guidelines but recognize that there may be some variation by location, as the guidelines are meant to be customized in each jurisdiction [14]. While a comprehensive assessment of health of SIVH would include other conditions such as chronic diseases, screening for these conditions are based on clinical judgement and/or state/clinic-based policies; hence, they are not routinely screened for during the domestic medical examination. Sites did not share these data.

For TB, information on diagnosis was reported and categorized as no evidence of TB, clinically active TB disease (person had clinical, bacteriological, and/or radiographic evidence of current pulmonary TB), not clinically active TB disease (person with a history of previous episode[s] of TB or abnormal stable radiographic findings and had a positive reaction to tuberculin skin test [TST], negative cultures, and no clinical and/or radiographic evidence of current disease), or latent tuberculosis infection (LTBI; person had a positive interferon gamma release assay or TST and negative diagnostic workup for TB) [15]. For hepatitis B, hepatitis B virus surface antigen (HBsAg), antibody to hepatitis B core antigen (anti-HBc), and antibody to hepatitis B surface antigen (anti-HBs) results were used to categorize a person’s hepatitis B virus (HBV) infection status. Status was categorized as susceptible (HBsAg, anti-HBc, and anti-HBs all negative), uninfected/susceptibility unknown (HBsAg negative, anti-HBc and anti-HBs unknown), infected (HBsAg positive), immune through natural infection (HBsAg negative, anti-HBc positive, and anti-HBs positive), immune through hepatitis B (hepB) vaccination (HBsAg negative, anti-HBc negative, and anti-HBs positive), and immune but not specified (HBsAg negative, anti-HBs positive, and anti-HBc unknown) [16]. During the time of data collection, CDC guidelines recommended screening blood for lead in children ages 6 months to 16 years, with EBLL defined as ≥5 mcg/dL [17]. For hepatitis C, malaria, strongyloidiasis, schistosomiasis, syphilis, gonorrhea, chlamydia, and HIV, the outcome was categorized as either “screened and positive” or “screened and negative.” Persons who were screened and had unknown results reported were excluded from the analysis.

### Data analysis

Frequencies and proportions were calculated to describe demographic characteristics and disease prevalence; results were stratified by SIV population (Afghan versus Iraqi) and age at screening visit (adult ≥18 years, child <18 years). We used χ^2^ tests to compare time of domestic medical examination and each disease condition by nationality of SIVH (Iraqi or Afghan). χ^2^ tests were also used to determine any differences in disease conditions between SIVH and refugees. *p*-Values were reported if frequency per cell was ≥5. Statistical significance was noted at a *p*-value <0.05. Denominators varied because of missing data and screening differences across sites. That is, nine sites provided screening data for TB, hepatitis B, hepatitis C, and lead; eight sites provided screening data for malaria, schistosomiasis, strongyloidiasis, intestinal parasites, and sexually transmitted infections. Thus, prevalence of health conditions was reported among those with available screening results. When calculating the proportions of persons who screened positive for a condition, we excluded sites that were only able to provide positive testing results without providing denominator data on how many people were screened for the condition, or who did not screen for or report a specific condition.

A modified Poisson regression was used to model the adjusted prevalence ratio (PR) (adjusting for age and sex) while accounting for state-level clustering. Nationality (Iraqi SIVH as reference) was the primary exposure variable, and LTBI, hepatitis B outcomes (susceptible, infected, and immune because of hepB vaccination), presence of at least one pathogenic parasite, and EBLL were the primary outcome variables. Medical conditions with five or fewer cases were excluded from the analyses. The prevalence of demographic characteristics and diseases among Iraqi and Afghan SIVH were compared to those of Iraqi and Afghan refugees (from same data source and time period) using χ^2^ analysis. A *p*-value of <0.05 was defined as statistically significant.

## Results

Of the 6,124 SIV adults included in our analysis, 1,112 (18.2%) were Iraqi and 5,012 (81.8%) were Afghan (Table 1). Of the 4,816 SIV children included in our analysis, 851 (17.7%) were Iraqi and 3,965 (82.3%) were Afghan. A majority of SIV adults were male (Iraqi 54.0%, Afghan 58.6%) and aged 18–44 (Iraqi 86.0%, Afghan 97.7%). More SIV children were male (Iraqi 56.2%, Afghan 52.2%) and aged 6–17 (Iraqi 50.2%, Afghan 40.7%). The average age of adults was 29.7 years (standard deviation [SD]: 7.6), and the average age for children was 5.6 years (SD: 4.4). The primary language spoken by or used by an interpreter for Iraqi SIVH was Arabic, and the primary languages spoken by or used by an interpreter for Afghan SIVH were Dari, Pashto, and Farsi. Before resettlement in the US, the majority of Iraqi and Afghan SIVH lived in their country of birth, while some Iraqis lived in Turkey or Jordan. About 97.4% of SIV adults and children had their domestic medical examination within 90 days of arrival in the US; this did not differ by nationality (Table 2).

**Table 1:** Demographic characteristics of adults and children who resettled to the US with Special Immigrant Visas (SIV), 2014–2016^a^.

Demographic Characteristics	Adults ≥18 years old			Children <18 years old		
	All	Iraq	Afghanistan	All	Iraq	Afghanistan
	*n* (%)	*n* (%)	*n* (%)	*n* (%)	*n* (%)	*n* (%)
**Total**	6,124	1,112 (18.2)	5,012 (81.8)	4,816	851 (17.7)	3,965 (82.3)
**Sex**						
Female	2,587 (42.3)	512 (46.0)	2,075 (41.4)	2,267 (47.1)	372 (43.7)	1,895 (47.8)
Male	3,537 (57.8)	600 (54.0)	2,937 (58.6)	2,548 (52.9)	478 (56.2)	2,070 (52.2)
**Age range (years)**						
0–2				1,463 (30.4)	198 (23.3)	1,265 (31.9)
3–5				1,313 (27.3)	226 (26.6)	1,087 (27.4)
6–17				2,040 (42.4)	427 (50.2)	1,613 (40.7)
18–44	5,850 (95.5)	956 (86.0)	4,894 (97.7)			
45–64	258 (4.2)	145 (13.0)	113 (2.3)			
≥65	16 (0.3)	11 (1.0)	5 (0.1)			
**Primary language**						
Dari	2,519 (41.1)	3 (0.3)	2,516 (50.2)	2,098 (43.6)	3 (0.4)	2,095 (52.8)
Pashto	874 (14.3)	0 (0)	874 (17.4)	947 (19.7)	0 (0)	947 (23.9)
Farsi	820 (13.4)	0 (0)	820 (16.4)	622 (12.9)	0 (0)	622 (12.9)
Arabic	889 (14.5)	843 (75.8)	46 (0.9)	720 (15)	703 (82.6)	17 (0.4)
Others^b^	1,036 (16.9)	266 (23.9)	770 (15.3)	431 (9)	145 (17.1)	286 (7.2)
**Country of last residence**^**c**^						
Afghanistan	3,849 (62.9)	3 (0.3)	3,846 (76.7)	3,293 (68.4)	0 (0)	3,293 (83.1)
Iraq	852 (13.9)	852 (76.6)	0 (0)	646 (13.4)	645 (75.8)	1 (0.03)
Turkey	56 (0.9)	50 (4.5)	6 (0.1)	42 (0.9)	39 (4.6)	3 (0.1)
Pakistan	30 (0.5)	0 (0)	30 (0.6)	11 (0.2)	0 (0)	11 (0.3)
Jordan	24 (0.4)	24 (2.2)	0 (0)	17 (0.4)	17 (2.0)	0 (0)
Others	66 (1.0)	14 (1.2)	49 (0.1)	39 (0.8)	15 (1.7)	23 (0.6)
Unknown	1,247 (20.4)	166 (14.9)	1,081 (21.6)	768 (15.9)	134 (15.8)	634 (16.0)

^a^Percentages may not add up to 100% because of rounding.

^b^Other languages include Uzbek, Kurdish, Armenian, English, and Urdu.

^c^A total of 1,247 SIV adults (Iraqi SIV: 166 [14.9%]; Afghan SIV: 1,081 [21.6%]) had missing information about country of last residence. A total of 768 SIV children (Iraqi SIV: 134 [15.8%]; Afghan SIV: 634 [16.0%]) had missing information about country of last residence.

Abbreviation: SIV, Special Immigrant Visa

**Table 2:** Domestic health screening results among adults and children who resettled to the US with Special Immigrant Visas (SIV), 2014–2016^a^^,^^b^^,^^c^.

Health Screening Results	Adults ≥18 years old				Children <18 years old			
	All	Iraq	Afghanistan	*p*-value	All	Iraq	Afghanistan	*p*-value
	*n* (%)	*n* (%)	*n* (%)		*n* (%)	*n* (%)	*n* (%)	
**Total**	6,124	1,112 (18.2)	5,012 (81.8)		4,816	851 (17.7)	3,965 (82.3)	
**Time of domestic medical examination**	*n* = 6063	*n* = 1,104	*n* = 4,959	0.54	*n* = 4,774	*n* = 843	*n* = 3,931	0.08
<30 days	3,281 (54.1)	580 (52.5)	2,701 (54.5)		2,764 (57.9)	466 (55.3)	2,298 (58.5)	
30–90 days	2,665 (44.6)	503 (45.6)	2,162 (43.6)		1,940 (40.6)	370 (43.9)	1,570 (39.9)	
>90 days	117 (1.9)	21 (1.9)	96 (1.9)		70 (1.5)	7 (0.8)	63 (1.6)	
**TB**^d^	*n* = 3471	*n* = 451	*n* = 3,020	0.01	*n* = 2,673	*n* = 320	*n* = 2,353	0.95
No evidence of TB	2,963 (85.4)	408 (90.7)	2,555 (84.6)		2,591 (96.9)	310 (96.9)	2,281 (96.9)	
Clinically active	2 (0.1)	0 (0)	2 (0.1)		0 (0)	0 (0)	0 (0)	
Not clinically active	8 (0.2)	1 (0.2)	7 (0.2)		0 (0)	0 (0)	0 (0)	
LTBI	498 (14.4)	42 (9.3)	456 (15.1)		82 (3.1)	10 (3.1)	72 (3.2)	
**Hepatitis B**^**e**^	*n* = 5777	*n* = 1,069	*n* = 4,708	<0.0001	*n* = 3,988	*n* = 741	*n* = 3,247	<0.0001
Susceptible	3,669 (63.5)	772 (72.2)	2,897 (61.5)		1,064 (26.7)	223 (30.1)	841 (25.9)	
Uninfected, susceptibility unknown	616 (10.7)	166 (15.5)	450 (9.6)		1,340 (33.6)	199 (26.9)	1,141 (35.1)	
Infected	106 (1.8)	6 (0.6)	100 (2.1)		23 (0.6)	1 (0.1)	22 (0.7)	
Immune								
Natural infection	331 (5.7)	15 (1.4)	316 (6.7)		33 (0.8)	3 (0.4)	30 (0.9)	
Hepatitis B vaccination	978 (16.9)	104 (9.7)	874 (18.6)		1,487 (37.3)	311 (42.0)	1,176 (36.2)	
Not specified	77 (1.3)	6 (0.6)	71 (1.5)		41 (1)	4 (0.5)	37 (1.1)	
**Hepatitis C**^f^	*n* = 4,007	*n* = 598	*n* = 3,409	N/A	*n* = 2,554	*n* = 342	*n* = 2,212	N/A
Screened, positive	31 (0.8)	4 (0.7)	27 (0.8)		8 (0.3)	2 (0.6)	6 (0.3)	
**Malaria**^g^	*n* = 1,572	*n* = 136	*n* = 1,436		*n* = 1,219	*n* = 83	*n* = 1,136	
Screened, positive	2 (0.1)	0 (0)	2 (0.1)		5 (0.4)	0 (0)	5 (0.4)	
**Strongyloidiasis**^h^	*n* = 2,578	*n* = 201	*n* = 2,377	0.6	*n* = 2,018	*n* = 131	*n* = 1,887	0.9
Screened, positive	75 (2.9)	7 (3.5)	68 (2.9)		50 (2.5)	3 (2.3)	47 (2.5)	
**Schistosomiasis**^h^	*n* = 130	*n* = 42	*n* = 88		*n* = 96	*n* = 17	*n* = 79	
Screened, positive	2 (1.5)	0 (0)	2 (2.3)		2 (2.1)	0 (0)	2 (2.5)	
**Other intestinal parasites**^i^	*n* = 3,982	*n* = 555	*n* = 3,427	0.3	*n* = 3,341	*n* = 451	*n* = 2,890	0.4
One or more intestinal parasites	1,235 (31.0)	183 (33.0)	1,052 (30.7)		737 (22.1)	93 (20.6)	644 (22.3)	
*Blastocystis*	435 (10.9)	46 (8.3)	389 (11.4)		233 (7.0)	22 (4.9)	211 (7.3)	
*Giardia*	111 (2.8)	10 (1.8)	101 (3.0)		272 (8.1)	17 (3.8)	255 (8.8)	
*Dientamoeba*	108 (2.7)	6 (1.1)	102 (3.0)		76 (2.3)	12 (2.7)	64 (2.2)	
*Ascaris*	60 (1.5)	0 (0)	60 (1.8)		45 (1.4)	0 (0)	45 (1.6)	
*Entamoeba histolytica*	74 (1.9)	9 (1.6)	65 (1.9)		25 (0.8)	2 (0.4)	23 (0.8)	
*Hymenolepsis*	3 (0.1)	0 (0)	3 (0.1)		17 (0.5)	0 (0)	17 (0.6)	
Others	2 (0.05)	0 (0)	2 (0.06)		5 (0.1)	0 (0)	5 (0.2)	
**Syphilis**^j^	*n* = 5,304	*n* = 928	*n* = 4,376	N/A	*n* = 723	*n* = 105	*n* = 618	N/A
Screened, positive	9 (0.2)	2 (0.2)	7 (0.2)		1 (0)	0 (0)	1 (0.2)	
**Gonorrhea**^k^	*n* = 18	*n* = 4	*n* = 14	N/A	*n* = 4	*n* = 2	*n* = 2	N/A
Screened, positive	0 (0)	0 (0)	0 (0)		0 (0)	0 (0)	0 (0)	
**Chlamydia**^k^	*n* = 1,573	*n* = 121	*n* = 1,452	N/A	*n* = 166	*n* = 15	*n* = 151	N/A
Screened, positive	14 (0.9)	2 (1.7)	12 (0.8)		2 (1.2)	0 (0)	2 (1.3)	
**HIV**^k^	*n* = 5,782	*n* = 1,027	*n* = 4,755	N/A	*n* = 3,924	*n* = 662	*n* = 3,262	N/A
Positive, screened/unscreened (type 1, type 2, or unknown)	4 (0.07)	1 (0.1)	3 (0.06)		1 (0.03)	1 (0.2)	0 (0)	
**Blood lead level** ^l^				N/A	*n* = 2,368	*n* = 543	*n* = 1,825	<0.0001
<5 mcg/dL	N/A	N/A	N/A		1,181 (49.9)	470 (86.6)	711 (39)	
5–9 mcg/dL	N/A	N/A	N/A		944 (39.9)	71 (13.1)	873 (47.8)	
10–19 mcg/dL	N/A	N/A	N/A		201 (8.5)	2 (0.4)	199 (10.9)	
20–44 mcg/dL	N/A	N/A	N/A		36 (1.5)	0 (0)	36 (2)	
45–69 mcg/dL	N/A	N/A	N/A		2 (0.1)	0 (0)	2 (0.1)	
70+ mcg/dL	N/A	N/A	N/A		4 (0.2)	0 (0)	4 (0.2)	

^a^Percentages may not add up to 100% because of rounding.

^b^Proportion of all SIVH who were not screened for a particular medical condition: latent TB (14.3%; 3% for Iraqi and Afghan adults, 35% for Iraqi children, and 27% for Afghan children); HBV (6.5%; 3% for Iraqi adults, 2% for Afghan adults, and 12% for Iraqi and Afghan children); hepatitis C virus (40.0%); malaria (74.4%); *Strongyloides* (57.9%); *Schistosoma* (97.9%), intestinal parasites (33.1%); syphilis (44.9%); gonorrhea (99.8%); chlamydia (84.1%); EBLL (77.5%); and HIV (17.6%).

^c^We used χ^2^ tests to compare characteristic or disease condition by nationality (Iraqi or Afghan). *p-*Values were reported if frequency per cell was ≥5. Statistical significance was noted at a *p*-value <0.05.

^d^For TB, information on diagnosis was reported and categorized as no evidence of TB, clinically active, not clinically active, and LTBI [15]. TB disease diagnosis was made by a positive smear, culture, or clinical diagnosis of pulmonary TB. A classification of not clinically active TB was made when a person had a history of previous episode(s) of TB or abnormal stable radiographic findings and had a positive reaction to TST, negative cultures, and no clinical and/or radiographic evidence of current disease. Diagnosis of LTBI was made by a positive interferon gamma release assay (IGRA) or TST and negative diagnostic workup for TB. The majority of adults (95%) were tested using IGRA. Among children tested for LTBI, 87% were tested using IGRA and 17% were tested using TST. Data were included if states provided information about TB diagnosis for an individual.

^e^HBV status was categorized as susceptible (HBsAg, anti-HBc, and anti-HBs all negative), uninfected/susceptibility unknown (HBsAg negative, anti-HBc and anti-HBs unknown), infected (HBsAg positive), immune through natural infection (HBsAg negative, anti-HBc positive, and anti-HBs positive), immune through hepatitis B vaccination (HBsAg negative, anti-HBc negative, and anti-HBs positive) and immune but not specified (HBsAg negative, anti-HBs positive, and anti-HBc unknown) [16]. About 3.5% of all SIV adults and 6% of all SIV children had HBV results where the interpretation was unknown or unclear.

^f^Hepatitis C was diagnosed by any of the following: detection of antibody to hepatitis C virus (anti-HCV), a positive recombinant immunoblot assay (RIBA) result, or a positive HCV RNA polymerase chain reaction (PCR) result.

^g^Malaria diagnosis was laboratory confirmed using either microscopy or by a rapid diagnostic test.

^h^*Strongyloides* and *Schistosoma* diagnoses were laboratory confirmed using either microscopy or by serology testing.

^i^Intestinal parasite infection diagnoses were laboratory confirmed using stool ova and parasite testing. Reported *p*-value compares Iraqi and Afghan SIVH with any intestinal parasite reported (pathogenic and nonpathogenic) to Iraqi and Afghan SIVH with no intestinal parasite reported.

^j^Syphilis diagnosis was made via a positive non-treponemal test (venereal disease research laboratory [VDRL] or rapid plasma reagin [RPR]) followed by a positive confirmatory treponemal test (e.g., *Treponema pallidum*-particle agglutination [TP-PA], microhemagglutination assay for *T*. *pallidum* [MHA-TP]). Syphilis testing is recommended in all persons ≥15 years of age if no overseas testing results are available, and in persons <15 years of age if sexually active.

^k^Gonorrhea, chlamydia, and HIV diagnoses were made via laboratory-confirmed testing.

^l^Blood lead level screening applies to children from 6 months up to 16 years of age only.

Abbreviations: anti-HBc, antibody to hepatitis B core antigen; anti-HBs, antibody to hepatitis B surface antigen; EBLL, elevated blood lead levels; HBsAg, hepatitis B virus surface antigen; HBV, hepatitis B virus; HCV, hepatitis C virus; LTBI, latent tuberculosis infection; SIV, Special Immigrant Visa; SIVH, SIV holder; TB, tuberculosis; TST, tuberculin skin test

### SIV adults

Overall, 2,963 (85.4%) SIV adults had no evidence of TB, and 14.4% were diagnosed with LTBI (Table 2). Afghan adults were more likely to have LTBI compared to Iraqi adults (PR: 2.0; 95% confidence interval [CI] 1.5–2.7) (Table 3). Approximately 63.5% of adults were susceptible to HBV infection (Iraqi 72.2%, Afghan 61.5%) (Table 2). However, 10.7% of all adults were uninfected with HBV with unknown susceptibility (Iraqi 15.5%, Afghan 9.6%); therefore, the proportion susceptible to hepatitis B may be higher. Afghan adults were less likely to be susceptible to HBV infection compared to Iraqi adults (PR: 0.8; 95% CI 0.7–0.8) (Table 3). About 1.8% of all SIV adults were infected with HBV (Iraqi 0.6%, Afghan 2.1%), while 23.9% were immune (Iraqi 11.7%, Afghan 26.8%) (Table 2). Afghan adults were more likely to be infected with HBV (PR: 4.6; 95% CI 3.6–6.0) but were also more likely to have vaccine-induced immunity to HBV infection (PR: 2.0; 95% CI 1.5–2.6) compared to Iraqi adults (Table 3). Three percent of Iraqi and Afghan SIV adults screened positive for *Strongyloides* (Table 2). Thirty-one percent of screened adults had at least one intestinal parasite. *Blastocystis* (10.9%), *Giardia* (2.8%), and *Dientamoeba* (2.7%) were the most common intestinal parasites found in both SIV populations. There was no difference in the prevalence of pathogenic intestinal parasites (excluding *Blastocystis* and *Dientamoeba*, which are considered controversial for treatment) between Afghan and Iraqi adults (PR: 1.6; 95% CI 1.0–2.6) (Table 3).

**Table 3:** Adjusted PRs for select medical conditions comparing Afghan and Iraqi Special Immigrant Visa Holders (SIVH) who resettled to the US, 2014–2016^a^.

Medical Conditions	Adults ≥18 years old	Children <18 years old
	aPR (95% CI) Ref: Iraqi SIVH	aPR (95% CI) Ref: Iraqi SIVH
**LTBI**	2.0 (1.5–2.7)	1.0 (0.4–2.7)
**Hepatitis B**^**b**^		
Susceptible	0.8 (0.7–0.8)	1.0 (0.7–1.3)
Infected	4.6 (3.5–6.0)	N/A
Immune through vaccination	2.0 (1.5–2.6)	1.0 (0.8–1.2)
**Pathogenic parasites**	1.6 (1.0–2.6)	2.7 (2.4–3.2)
**EBLL** (≥5 mcg/dL)	N/A	2.0 (1.5–2.5)

^a^Poisson regression was used to model the adjusted PRs (adjusted for age and sex) to assess association of nationality and outcomes. Iraqi nationality was used as reference.

^b^HBV status was categorized as susceptible (HBsAg, anti-HBc, and anti-HBs all negative), infected (HBsAg positive), and immune through hepatitis B vaccination (HBsAg negative, anti-HBc negative, and anti-HBs positive).

Abbreviations: anti-HBc, antibody to hepatitis B core antigen; anti-HBs, antibody to hepatitis B surface antigen; aPR, adjusted prevalence ratio; CI, confidence interval; EBLL, elevated blood lead levels; HBsAg, hepatitis B virus surface antigen; HBV, hepatitis B virus; LTBI, latent tuberculosis infection; Ref, reference; SIVH, SIV holder

### SIV children

Overall, 2,591 (96.9%) SIV children had no evidence of TB, and 3.1% had a diagnosis of LTBI (Table 2). Approximately 0.6% of all children were HBV infected, while 39.1% were immune (Iraqi 42.9%, Afghan 38.2%). Furthermore, about 26.7% of children were susceptible to HBV infection, although this proportion may be higher given that 33.6% of children were uninfected with their susceptibility unknown. Approximately 2.1% of children screened positive for *Strongyloides* infection, and 22.1% of children had at least one intestinal parasite. *Giardia* (8.1%), *Blastocystis* (7.0%), and *Dientamoeba* (2.3%) were the most common intestinal parasites found. Afghan children were more likely to have pathogenic intestinal parasites (excluding *Blastocystis* and *Dientamoeba*) compared to Iraqi children (PR: 2.7; 95% CI 2.4–3.2) (Table 3). Over half of all SIV children had an EBLL, with higher prevalence among Afghans than among Iraqis (PR: 2.0; 95% CI 1.5–2.5).

### SIVH versus refugees

Iraqi and Afghan SIVH were compared to refugees with the same nationality (Iraqi 9,368; Afghan 1,407). Afghan SIV adults (17.8%) were less likely to have LTBI than were Afghan refugee adults (26.1%) (χ^2^ *p* < 0.0001), and Afghan SIV children (39.9%) were less likely to be susceptible to HBV infection compared to Afghan refugee children (52.8%) (χ^2^ *p* < 0.0001). Afghan SIV adults (30.7%) and children (22.3%) had higher prevalence of at least one intestinal parasite (other than *Strongyloides* or schistosomiasis) than their respective Afghan refugee populations (adults 12.5%, children 13.4%) (χ^2^ *p* < 0.0001). Iraqi SIV children (41.1%) were less likely to be susceptible to hepatitis B than were Iraqi refugee children (47.2%) (χ^2^ *p* = 0.01). Iraqi SIV adults (33.0%) had higher prevalence of at least one intestinal parasite relative to Iraqi refugee adults (25.9%) (χ^2^ *p* = 0.0004). The prevalence of at least one intestinal parasite and EBLL did not differ between SIV and refugee children from both Iraq and Afghanistan.

### SIVH not screened for a particular condition

The proportions of all SIVH who were not screened for a particular medical condition are LTBI (14.3%), HBV (6.5%), hepatitis C virus (HCV) (40.0%), malaria (74.4%), *Strongyloides* (57.9%), *Schistosoma* (97.9%), intestinal parasites (33.1%), syphilis (44.9%), gonorrhea (99.8%), chlamydia (84.1%), EBLL (77.5%), and HIV (17.6%). These are crude estimates only and not adjusted for age, sex, or nationality.

## Discussion

In this analysis, we described the health of SIV populations after arrival into the US, focusing on conditions typically assessed at the domestic medical examination according to CDC’s *Guidelines for the US Domestic Medical Examination for Newly Arrived Refugees*. Key findings were reported on the health of 10,940 SIVH from Iraq and Afghanistan who received the domestic medical examination in nine regions in the US. First, about 14% of adults had LTBI, with a higher prevalence in Afghans than in Iraqis. Second, the majority of adults were susceptible to HBV infection, with Iraqis more likely than Afghans to be susceptible (i.e., no evidence of immunity due to prior infection or vaccination). Third, about 27% of SIVH had at least one intestinal parasite infection. Finally, over half of all SIV children had EBLL; the prevalence among Afghan children (61%) was higher than the prevalence among Iraqis.

While published data on LTBI prevalence among Afghan populations in general are limited, LTBI prevalence estimates in Iraqi populations range from 0.9% to 14.1% [8–10]. The incidence of TB disease in Afghanistan is 189 per 100,000 people compared to 42 per 100,000 people in Iraq [18,19], which may explain our finding of higher prevalence of LTBI among Afghans. Domestic clinicians screening newly arrived SIVH should refer to CDC’s *Guidelines for the US Domestic Medical Examination for Newly Arrived Refugees* to ensure they are appropriately screening for and treating LTBI, with special attention to adults, who are not routinely tested for *Mycobacterium tuberculosis* infection in the overseas medical exam [20].

About 2% of SIV adults and 1% of SIV children presented with chronic HBV infection, while the majority of SIV adults and a quarter of SIV children were susceptible to HBV infection. Multiple studies have documented estimates of chronic HBV infection in predominantly refugee and migrant Iraqi populations ranging from 0.4% to 2.5% [8,10,21–24]. Chronic HBV infection estimates in Afghan populations range from 1.5% to 9.2% [23;25–30], with higher burden in refugees [25,27]. Other studies similarly showed that susceptibility to hepatitis B among resettled adult Iraqi populations was over 70% [23,31] and over 50% among adult Afghan populations [31].

While hepatitis B vaccination was introduced in Iraq in 1985 to all children and adults with high-risk conditions, routine vaccination has been interrupted by war and military action in the early part of the 21st century [32]. Through the WHO Expanded Program on Immunization, the hepatitis B vaccine birth dose was introduced in Iraq in 2004, and infants were eligible to receive all three doses of the vaccine [33]. Hepatitis B vaccination was introduced in Afghanistan in 2006, while the birth dose was introduced only in 2014 [33]. In 2017, WHO estimated that 63% of Iraqi infants and 65% of Afghan infants received all three doses of the vaccine [33]. It is unclear why at least a third of Iraqi children and a quarter of Afghan children in our analysis were susceptible to hepatitis B given that hepatitis B vaccination should be administered (or evidence of previous vaccination documented) to all SIV children up through age 18 via routine childhood immunization programs or during the overseas predeparture screening exam per the Vaccination Technical Instructions [7]. Possible explanations for hepatitis B susceptibility among SIV children could include lack of overseas vaccine availability, recent administration of the vaccine (leaving insufficient time for detectable antibody levels to develop), the more recent introduction of hepatitis B vaccine in Afghanistan, inadequate education of parents about vaccination importance, inappropriately timed or missed immunizations, and inaccurate immunization cards [34].

Similar to our analysis, other publications report that vaccine-induced immunity to hepatitis B is lower for the Iraqi adult population (5%) than for the Afghan population (40%) [31]. In Iraq, the hepatitis B vaccine is given in adulthood only to high-risk adults, so it is possible that the vaccine was not given to most Iraqi adults while they were in Iraq. Another explanation for the low prevalence of vaccine-induced immunity to HBV among both Iraqi and Afghan populations is that SIV families who go into hiding while serving the US government or military abroad may have limited access to healthcare and preventive health services [12,13].

Unlike refugees, SIVH are not currently eligible to participate in the voluntary Vaccination Program for US-bound Refugees, which was created to provide 1–2 doses of certain vaccines, including hepatitis B, overseas [35]. However, as mentioned previously, SIV children through age 18 applying for US residency are required to receive the first dose of the hepatitis B vaccine series to comply with immigration requirements [7], whereas SIV adults are unlikely to receive hepatitis B vaccine. The overseas vaccination program for refugees is variably implemented across different sites in the Middle East, which could possibly explain why SIV Iraqi and Afghan children are less susceptible to HBV infection compared to US-bound Iraqi and Afghan refugee children, who may have not received doses of the hepatitis B vaccine series. Both Iraq and Afghanistan are countries with intermediate hepatitis B endemicity (2% to 7% of the population with chronic hepatitis B infection), and the prevalence of HBV infection susceptibility is high among SIVH. US clinicians caring for Iraqi and Afghan SIVH should continue hepatitis B screening for all adults and children, and offer vaccinations to those susceptible to infection, per CDC’s *Guidelines for the US Domestic Medical Examination for Newly Arriving Refugees*: *Screening for Viral Hepatitis During the Domestic Medical Examination of Newly Arrived Refugees* [36,37].

The most common intestinal parasites seen in both SIV adults and children included *Blastocystis*, *Giardia*, and *Dientamoeba*. Studies in Iraqi populations report a high overall prevalence of gastrointestinal infections with estimates up to 64% of the population, and commonly reported parasites include *Blastocystis* (32%–36%) [8, 38], *Giardia* (28%–45%) [8,38–41], and *Entamoeba* (23%) [39]. In Afghan populations, overall prevalence of intestinal parasites as high as 39% has been noted [42], and commonly reported parasites include *Giardia* (15%–59%) [42–44] and *Ascaris* (20%–35%) [41]. *Blastocystis* was found in 15% of Polish soldiers returning from peacekeeping missions in both Afghanistan and Iraq; the infections were presumably contracted in these regions [45]. Several parasites may not cause symptoms; however, others have the potential to cause complications. SIVH, unlike Iraqi and Afghan refugees, do not receive the predeparture parasite treatment as part of CDC’s overseas presumptive treatment program. Therefore, US clinicians caring for SIVH during the domestic medical examination should strongly consider screening and treating for intestinal parasites according to CDC’s *Guidelines for the US Domestic Medical Examination for Newly Arriving Refugees*: *Domestic Intestinal Parasite Guidelines* to prevent further transmission or complications [46].

Few SIVH from Iraq and Afghanistan were reported positive for any of the following: syphilis, gonorrhea, chlamydia, or HIV. However, the majority of SIVH were not screened for gonorrhea and chlamydia. The CDC’s *Guidelines for the US Domestic Medical Examination for Newly Arriving Refugees*: *Screening for Sexually Transmitted Diseases during the Domestic Medical Examination for Newly Arrived Refugees* recommend screening for certain sexually transmitted infections based on whether overseas screening was done (for syphilis), symptoms, site-based clinical screening policies for these conditions, age, and other risk factors [47].

Half of all SIV children had a blood lead level of 5 mcg/dL or greater, with 61% of Afghan children screening positive for EBLL and 13.2% recording a blood lead level above 10 mcg/dL. One study among resettled Afghan refugee children similarly revealed prevalence of EBLL up to 55% [48]. There have been few reports of lead exposure in Afghan children, and these identified the use of lead-containing eye cosmetics, a common cultural practice among even very young children and babies from Afghanistan and several Middle Eastern and African countries [48], as the source of lead exposure [49,50]. Studies among resettled Iraqi refugee children show that about 20% of children (versus 13% in our analysis) have blood lead levels over 5 mcg/dL [9, 49], while about 1% to 3% of children have levels above 10 mcg/dL [8,10,51]. While potential lead exposures among SIV children were not evaluated in our analysis, US clinicians should ensure that all SIV children 16 years of age and under are evaluated at the domestic medical examination and managed for lead poisoning and malnutrition (including an evaluation for iron deficiency) after arrival [17]. Management includes discussing potential lead exposures with parents of those children with higher levels and retesting blood lead levels of all children 6 years of age and under within 3–6 months after resettlement [17].

This analysis has some limitations. First, health screening data were not collected and reported uniformly across all nine sites; therefore, denominators across medical conditions and diagnoses vary. For example, *Blastocystis hominis* was reported as a pathogenic parasite by some clinicians and a nonpathogenic parasite by other clinicians (treatment for controversial parasites, such as *Blastocystis* and *Dientamoeba*, is considered if there are no other explanations for symptoms [46]). To account for this variation, for states that diagnosed *Blastocystis* or *Dientamoeba* only in an SIVH but reported the parasite as pathogenic, we excluded these data in the calculation of the adjusted PR for pathogenic parasites. Second, the CDC’s *Guidelines for the US Domestic Medical Examination for Newly Arriving Refugees* differentiate screening according to nationality, age of patient, and documented overseas test results and interventions; thus, not all tests were conducted for all SIVH. Third, it is possible that clinicians could misclassify Iraqi and Afghan SIVH as refugees. Hence, disease estimates could be underestimated or overestimated. Fourth, given that about 11% of SIV adults and 34% of SIV children were uninfected but had unknown susceptibility to HBV, the estimates for HBV susceptibility and immunity could be overestimated or underestimated. Fifth, sites only shared whether SIVH screened positive or negative for different infections such as HCV infection, but the test used was not reported. With HCV infection, it is possible that a person can test positive for HCV antibody but have undetectable levels of HCV RNA, indicating that a person may not be currently infected. With *Schistosoma*, a patient may test positive via serology but test negative via a stool (or urine) test, which also may not indicate current infection. Stool microscopy has a low sensitivity for *Strongyloides* and may not capture all cases. Another example is that *Entamoeba histolytica* cannot be easily distinguished from *Entamoeba dispar*, a nonpathogenic parasite, via stool microscopy. Therefore, based on the screening test used, the estimates for these infections reported in this article may be underestimated or overestimated. Sixth, there may be site-based differences that may impact screening outcomes that were not taken into account in our analysis. These differences include availability of transportation for SIVH, culturally competent translators, funding, and state-based health insurance policies. Lastly, given that data from SIVH who did not attend the domestic medical examination were not obtained, we were unable to determine if there were differences in disease estimates between SIVH who elected benefits versus those who did not, and those who attended the domestic medical examination versus those who did not.

To the authors’ knowledge, this is the first analysis of select communicable diseases and blood lead levels from the domestic medical examination among Iraqi and Afghan SIV populations and one of few to explore the health of these populations after resettlement to the US. Given that sites collected and shared data differently, and we did not capture detailed information on method or type of testing for most conditions, our findings only reflect those sites contributing data and not all SIV populations in the US.

In conclusion, we observed that 14% of SIV adults had LTBI, 27% of SIVH had at least one intestinal parasite, and about half of SIV children had EBLL. Most adults were susceptible to HBV. In general, prevalence of infection was higher for most conditions among Afghan SIVH compared to Iraqi SIVH. CDC’s *Guidelines for the US Domestic Medical Examination for Newly Arrived Refugees* can assist state public health departments and clinicians in the care of refugees and others receiving refugee benefits (including SIVH) during the routine domestic medical examination. Based on the results of this analysis, clinicians can ensure that SIVH receive the appropriate screening procedures and followed up with as necessary, paying particular attention to Afghan SIVH given their increased risk of several communicable diseases and EBLL (among children). Clinicians should also be aware that Iraqi and Afghan SIV populations differ in socioeconomic and migration histories from refugee populations from the same countries. Furthermore, access to overseas interventions and vaccination requirements differ between SIV and US-bound refugee populations. This could in part explain differences in disease estimates between SIV and refugee populations noted in this analysis. Future analyses can explore other aspects of health among resettled SIV populations, including noncommunicable diseases, vaccination coverage, mental health conditions, and long-term health outcomes.

## Supporting information

S1 STROBE Checklist STROBE, Strengthening the Reporting of Observational Studies in Epidemiology. (DOCX)

## Abbreviations

anti-HBc: antibody to hepatitis B core antigen
anti-HBs: antibody to hepatitis B surface antigen
CDC: Centers for Disease Control and Prevention
CI: confidence interval
DS: Department of State
EBLL: elevated blood lead levels
EDN: Electronic Disease Notification
HBsAg: hepatitis B virus surface antigen
HBV: hepatitis B virus
HCV: hepatitis C virus
IRB: Institutional Review Board
LTBI: latent tuberculosis infection
PR: prevalence ratio
SD: standard deviation
SIV: Special Immigrant Visa
SIVH: SIV holder
STROBE: Strengthening the Reporting of Observational Studies in Epidemiology
TB: tuberculosis
TST: tuberculin skin test
USRAP: US Refugee Admissions Program
