# Supplementary material for: Health of Special Immigrant Visa holders from Iraq and Afghanistan after arrival into the United States using Domestic Medical Examination data, 2014–2016: A cross-sectional analysis
Source: PLoS Med. 2020 Mar 31;17(3):e1003083. doi: 10.1371/journal.pmed.1003083 (PMC7108690; doi:10.1371/journal.pmed.1003083)
Supplement: S1 STROBE Checklist — (DOCX) [file pmed.1003083.s001.docx]

STROBE Statement—Checklist of items that should be included in reports of ***cross-sectional studies***

(accompanying excerpts are noted in a table below this section)

|  | Item No | Recommendation | Section, Paragraph # |
| --- | --- | --- | --- |
| **Title and abstract** | 1 | (*a*) Indicate the study’s design with a commonly used term in the title or the abstract | Abstract; 2 |
|  |  | (*b*) Provide in the abstract an informative and balanced summary of what was done and what was found | Abstract; 2 |
| Introduction | | | |
| Background/rationale | 2 | Explain the scientific background and rationale for the investigation being reported | Introduction; 1-3 |
| Objectives | 3 | State specific objectives, including any prespecified hypotheses | Introduction; 3 |
| Methods | | | |
| Study design | 4 | Present key elements of study design early in the paper | Methods; 1 |
| Setting | 5 | Describe the setting, locations, and relevant dates, including periods of recruitment, exposure, follow-up, and data collection | Methods; 1 |
| Participants | 6 | (*a*) Give the eligibility criteria, and the sources and methods of selection of participants | Methods; 1-2 |
| Variables | 7 | Clearly define all outcomes, exposures, predictors, potential confounders, and effect modifiers. Give diagnostic criteria, if applicable | Methods; 2-3 |
| Data sources/ measurement | 8* | For each variable of interest, give sources of data and details of methods of assessment (measurement). Describe comparability of assessment methods if there is more than one group | Methods; 2,4 |
| Bias | 9 | Describe any efforts to address potential sources of bias | Methods; 4 |
| Study size | 10 | Explain how the study size was arrived at | Methods; 1,4 |
| Quantitative variables | 11 | Explain how quantitative variables were handled in the analyses. If applicable, describe which groupings were chosen and why | Methods; 3,4 |
| Statistical methods | 12 | (*a*) Describe all statistical methods, including those used to control for confounding | Methods; 4,5 |
|  |  | (*b*) Describe any methods used to examine subgroups and interactions | Methods; 4,5 |
|  |  | (*c*) Explain how missing data were addressed | Methods; 4 |
|  |  | (*d*) If applicable, describe analytical methods taking account of sampling strategy | N/A |
|  |  | (*e*) Describe any sensitivity analyses | N/A |
| Results | | | |
| Participants | 13* | (a) Report numbers of individuals at each stage of study—eg numbers potentially eligible, examined for eligibility, confirmed eligible, included in the study, completing follow-up, and analysed | Results; 1 |
|  |  | (b) Give reasons for non-participation at each stage | N/A |
|  |  | (c) Consider use of a flow diagram | N/A |
| Descriptive data | 14* | (a) Give characteristics of study participants (eg demographic, clinical, social) and information on exposures and potential confounders | Results; 1, Table 1 |
|  |  | (b) Indicate number of participants with missing data for each variable of interest | Results; Table 2 |
| Outcome data | 15* | Report numbers of outcome events or summary measures | Results; 2-5, Tables 2-3 |
| Main results | 16 | (*a*) Give unadjusted estimates and, if applicable, confounder-adjusted estimates and their precision (eg, 95% confidence interval). Make clear which confounders were adjusted for and why they were included | Results; 2-3, Table 3 |
|  |  | (*b*) Report category boundaries when continuous variables were categorized | N/A |
|  |  | (*c*) If relevant, consider translating estimates of relative risk into absolute risk for a meaningful time period | N/A |
| Other analyses | 17 | Report other analyses done—eg analyses of subgroups and interactions, and sensitivity analyses | Results; 4 |
| Discussion | | | |
| Key results | 18 | Summarise key results with reference to study objectives | Discussion; 1 |
| Limitations | 19 | Discuss limitations of the study, taking into account sources of potential bias or imprecision. Discuss both direction and magnitude of any potential bias | Discussion; 10 |
| Interpretation | 20 | Give a cautious overall interpretation of results considering objectives, limitations, multiplicity of analyses, results from similar studies, and other relevant evidence | Discussion; 2-9 |
| Generalisability | 21 | Discuss the generalisability (external validity) of the study results | Discussion; 11 |
| Other information | | | |
| Funding | 22 | Give the source of funding and the role of the funders for the present study and, if applicable, for the original study on which the present article is based | Methods; 1 |

*Give information separately for exposed and unexposed groups.

**Note:** An Explanation and Elaboration article discusses each checklist item and gives methodological background and published examples of transparent reporting. The STROBE checklist is best used in conjunction with this article (freely available on the Web sites of PLoS Medicine at http://www.plosmedicine.org/, Annals of Internal Medicine at http://www.annals.org/, and Epidemiology at http://www.epidem.com/). Information on the STROBE Initiative is available at [www.strobe-statement.org](http://www.strobe-statement.org).

| **Item number** | **Description** | **Manuscript Excerpt** |
| --- | --- | --- |
| 1 | 1. Indicate the study’s design with a commonly used term in the title or the abstract 2. Provide in the abstract an informative and balanced summary of what was done and what was found | ‘This cross-sectional analysis included data from Iraqi and Afghan SIVH who received a domestic medical examination from January 2014 to December 2016.’  ‘We investigated the frequency and proportion of diseases and whether there were any differences in selected disease prevalence in SIVH from Iraq compared to SIVH from Afghanistan.’ |
| 2 & 3 | Explain the scientific background and rationale for the investigation being reported.  State specific objectives, including any prespecified hypotheses | **Presented throughout introduction.  ‘There is little information about health conditions in SIV populations despite the high number who have elected USRAP benefits.’  ‘Increasing clinician knowledge about common health conditions encountered in SIVH, including differences between Iraqi and Afghan SIVH, may facilitate diagnostic screening, physical examination, and referrals to additional healthcare providers in the United States. To the authors’ knowledge, this information is not currently available. Thus, we sought to describe health characteristics of recently arrived SIVH from Iraq and Afghanistan who were seen for domestic medical examinations.’  ‘Specifically, we investigated the frequency and proportion of diseases commonly screened for in the domestic medical examination and for certain diseases, whether there were any differences in prevalence for SIVH from Iraq compared to SIVH from Afghanistan.’ |
| 4 | Present key elements of study design early in the paper | ‘This cross-sectional analysis included data from Iraqi and Afghan SIVH who received a domestic medical examination between January 2014 and December 2016.’ |
| 5 | Describe the setting, locations, and relevant dates, including periods of recruitment, exposure, follow-up, and data collection | ‘… Iraqi and Afghan SIVH who received a domestic medical examination between January 2014 and December 2016. CDC collaborated with nine partners as part of a cooperative agreement (*CK12-1205*) to collect domestic screening exam data for analysis. Partners included the state refugee health programs in California, Colorado, Illinois, Kentucky, Minnesota, New York, and Texas; local partners in Marion County, Indiana; and an academic health center in Philadelphia, Pennsylvania.’ |
| 6 | (*a*) Give the eligibility criteria, and the sources and methods of selection of participants | ‘…included data Iraqi and Afghan SIVH who received a domestic medical examination between January 2014 and December 2016.’  ‘Data were obtained from partner refugee health databases. Some partners obtained individual-specific data from external programs (e.g., tuberculosis or lead prevention) in their jurisdictions.’  ‘Demographic information (sex, age, and primary language spoken by the SIVH or used by the interpreter during the exam), nationality, and country of last residence were either provided by the site, or, if a unique identifier was provided in the data set, obtained from matched records in CDC’s Electronic Disease Notification (EDN) system.’ |
| 7 | Clearly define all outcomes, exposures, predictors, potential confounders, and effect modifiers. Give diagnostic criteria, if applicable | ‘Demographic information (sex, age, and primary language spoken by the SIVH or used by the interpreter during the exam), nationality, and country of last residence were either provided by the site, or, if a unique identifier was provided in the data set, obtained from matched records in CDC’s Electronic Disease Notification (EDN) system.’  ‘Screening test results were collected for tuberculosis, hepatitis B, hepatitis C, malaria, strongyloidiasis, schistosomiasis, presence of other intestinal parasites, syphilis, gonorrhea, chlamydia, human immunodeficiency virus (HIV), and elevated blood lead levels (EBLL).  ‘For tuberculosis, information on diagnosis was reported and categorized as no evidence of TB…’  ‘For hepatitis C, malaria, strongyloidiasis, schistosomiasis, syphilis, gonorrhea, chlamydia, and HIV, we categorized the outcome as either ‘screened and positive’ or ‘screened and negative.’ Persons who were screened and had unknown results reported were excluded from the analysis.’ |
| 8 | For each variable of interest, give sources of data and details of methods of assessment (measurement). Describe comparability of assessment methods if there is more than one group | Most data were ‘… obtained from matched records in CDC’s Electronic Disease Notification (EDN) system. EDN is a centralized reporting system that notifies US state and local health departments and screening clinics of the arrival of immigrants with health conditions requiring medical follow-up, including tuberculosis-related conditions, and all refugees.’  ‘…results were stratified by SIV population (Afghan vs. Iraqi) and age at screening visit (adult ≥18 years, child <18 years).’ |
| 9 | Describe any efforts to address potential sources of bias | ‘Denominators varied because of missing data and screening differences across sites…Thus, prevalence of health conditions was reported among those with available screening results. When calculating the proportions of persons who screened positive for a condition, we excluded sites that were only able to provide positive testing results without providing denominator data on how many people were screened for the condition, or who didn’t screen for or report a specific condition.’ |
| 10 | Explain how the study size was arrived at | This analysis ‘…included data Iraqi and Afghan SIVH who received a domestic medical examination between January 2014 and December 2016. CDC collaborated with nine partners as part of a cooperative agreement…’  ‘Denominators varied because of missing data and screening differences across sites. Thus, prevalence of health conditions was reported among those with available screening results.’ |
| 11 | Explain how quantitative variables were handled in the analyses. If applicable, describe which groupings were chosen and why | ‘For tuberculosis, information on diagnosis was reported and categorized as no evidence of TB, clinically active TB disease (person had clinical, bacteriological, and/or radiographic evidence of current pulmonary TB), not clinically active TB disease…For hepatitis B…results were used to categorize a person’s hepatitis B virus (HBV) infection status. Status was categorized as susceptible (HBsAg, anti-HBc, and anti-HBs all negative)….’  ‘For hepatitis C, malaria, strongyloidiasis, schistosomiasis, syphilis, gonorrhea, chlamydia, and HIV, we categorized the outcome as either ‘screened and positive’ or ‘screened and negative.’  ‘…results were stratified by SIV population (Afghan vs. Iraqi) and age at screening visit (adult ≥18 years, child <18 years).’ |
| 12 | (*a*) Describe all statistical methods, including those used to control for confounding (*b*) Describe any methods used to examine subgroups and interactions  (*c*) Explain how missing data were addressed | **Described in detail under ‘Data Analysis’ on Page 9.  ‘Frequencies and proportions were calculated to describe demographic characteristics and disease prevalence; results were stratified by SIV population (Afghan vs. Iraqi) and age at screening visit (adult ≥18 years, child <18 years)…Poisson regression was used to model the adjusted prevalence ratio (adjusting for age and sex) while accounting for state-level clustering. Nationality (Iraqi SIVH as reference) was the primary exposure variable, and LTBI, hepatitis B outcomes (susceptible, infected, and immune because of hepB vaccination), presence of at least one pathogenic parasite, and EBLL were the primary outcome variables. Medical conditions with five or fewer cases were excluded from the analyses. The prevalence of demographic characteristics and diseases among Iraqi and Afghan SIVH were compared to those of Iraqi and Afghan refugees (from same data source and time period) using chi-square analysis. A p-value of <0.05 was defined as statistically significant.’  ‘Denominators varied because of missing data and screening differences across sites. Thus, prevalence of health conditions was reported among those with available screening results.’ |
| 13 | (a) Report numbers of individuals at each stage of study—eg numbers potentially eligible, examined for eligibility, confirmed eligible, included in the study, completing follow-up, and analysed | ‘Of the 6,124 SIV adults included in our analysis, 1,112 (18%) were Iraqi and 5,012 (82%) were Afghan (Table 1).’ |
| 14 | 1. Give characteristics of study participants (eg demographic, clinical, social) and information on exposures and potential confounders 2. Indicate number of participants with missing data for each variable of interest | ‘Of the 6,124 SIV adults included in our analysis, 1,112 (18.2%) were Iraqi and 5,012 (81.8%) were Afghan (Table 1). Of the 4,816 SIV children included in our analysis, 851 (17.7%) were Iraqi and 3,965 (82.3%) were Afghan. A majority of SIV adults were male (Iraqi 54.0%, Afghan 58.6%) and aged 18–44 (Iraqi 86.0%, Afghan 97.7%). More SIV children were male (Iraqi 56.2%, Afghan 52.2%) and aged 6–17 (Iraqi 50.2%, Afghan 40.7%). The average age of adults was 29.7 years (standard deviation [SD]: 7.6), and the average age for children was 5.6 years (SD: 4.4). The primary language spoken by or used by an interpreter for Iraqi SIVH was Arabic, and the primary languages spoken by or used by an interpreter for Afghan SIVH were Dari, Pashto, and Farsi. Before resettlement in the United States, the majority of Iraqi and Afghan SIVH lived in their country of birth, while some Iraqis lived in Turkey or Jordan. About 97.4% of SIV adults and children had their domestic medical examination within 90 days of arrival in the United States; this did not differ by nationality (Table 2).’  For the number of participants with missing data for each variable of interest, this information is located in detail in Table 2. |
| 15 | Report numbers of outcome events or summary measures | ‘Overall, 2,963 (85.4%) SIV adults had no evidence of tuberculosis, and 14.4% were diagnosed with LTBI.’  ‘Approximately 63.5% of adults were susceptible to HBV infection (Iraqi 72.2%, Afghan 61.5%). However, 10.7% of all adults were uninfected with HBV with unknown susceptibility (Iraqi 15.5%, Afghan 9.6%);…’  **See reports throughout the results section, summarized by disease conditions and nationality. |
| 16 | Give unadjusted estimates and, if applicable, confounder-adjusted estimates and their precision (eg, 95% confidence interval). Make clear which confounders were adjusted for and why they were included | ‘Afghan adults were more likely to have LTBI compared to Iraqi adults (PR: 2.0; 95% CI: 1.5–2.7)’  ‘Afghan adults were less likely to be susceptible to HBV infection compared to Iraqi adults (PR: 0.8; 95% CI: 0.7–0.8).’  ‘There were no differences in the prevalence of pathogenic intestinal parasites (excluding *Blastocystis* and *Dientamoeba,* which are considered controversial for treatment) between Afghan and Iraqi adults (PR: 1.6; 95% CI: 1.0–2.6).’  **Find additional information throughout results section and in Table 3. |
| 17 | Report other analyses done—eg analyses of subgroups and interactions, and sensitivity analyses | *SIVH vs. Refugees*  ‘Iraqi and Afghan SIVH were compared to refugees with the same nationality (Iraqi 9,368, Afghan 1,407). Afghan SIV adults (17.8%) were less likely to have LTBI than were Afghan refugee adults (26.1%) (χ^2^ p < 0.0001), and Afghan SIV children (39.9%) were less likely to be susceptible to hepatitis B virus infection compared to Afghan refugee children (52.8%) (χ^2^ p < 0.0001). Afghan SIV adults (30.7%) and children (22.3%) had higher prevalence of at least one intestinal parasite (other than *Strongyloides* or schistosomiasis) than their respective Afghan refugee populations (adults 12.5%, children 13.4%) (χ^2^ p < 0.0001). Iraqi SIV children (41.1%) were less likely to be susceptible to hepatitis B than were Iraqi refugee children (47.2%) (χ^2^ p = 0.01). Iraqi SIV adults (33.0%) had higher prevalence of at least one intestinal parasite relative to Iraqi refugee adults (25.9%)(χ^2^ p = 0.0004). The prevalence of at least one intestinal parasite and EBLL did not differ between SIV and refugee children from both Iraq and Afghanistan.’ |
| 18 | Summarise key results with reference to study objectives | ‘In this analysis, we described the health of SIV populations after arrival into the US, focusing on conditions typically assessed at the domestic medical examination according to CDC’s *Guidelines for the US Domestic Medical Examination for Newly Arrived Refugees.* Key findings were reported on the health of 10,940 SIVH from Iraq and Afghanistan who received the domestic medical examination in nine regions in the United States. First, about 14% of adults had LTBI, with a higher prevalence in Afghans than in Iraqis. Second, the majority of adults were susceptible to HBV infection, with Iraqis more likely than Afghans to be susceptible (i.e., no evidence of immunity due to prior infection or vaccination). Third, about 27% of SIVH had at least one intestinal parasite infection. Finally, over half of all SIV children had EBLL; the prevalence among Afghan children (61%) was higher than the prevalence among Iraqis.’ |
| 19 | Discuss limitations of the study, taking into account sources of potential bias or imprecision. | ‘This analysis has some limitations. First, health screening data were not collected and reported uniformly across all nine sites; therefore, denominators across medical conditions and diagnoses vary. For example, *Blastocystis hominis* was reported as a pathogenic parasite by some clinicians and a non-pathogenic parasite by other clinicians (treatment for controversial parasites, such as *Blastocystis* and *Dientamoeba,* is considered if there are no other explanations for symptoms [46])…Third, it is possible that clinicians could misclassify Iraqi and Afghan SIVH as refugees. Hence, disease estimates could be underestimated or overestimated. Fourth, given that about 11% of SIV adults and 34% of SIV children were uninfected but had unknown susceptibility to HBV, the estimates for HBV susceptibility and immunity could be overestimated or underestimated...’ |
| 20 | Give a cautious overall interpretation of results considering objectives, limitations, multiplicity of analyses, results from similar studies, and other relevant evidence | ‘While published data on LTBI prevalence among Afghan populations in general are limited, LTBI prevalence estimates in Iraqi populations range from 0.9% to 14.1% [8–10]. The incidence of tuberculosis disease in Afghanistan is 189 per 100,000 people compared to 42 per 100,000 people in Iraq [18, 19], which may explain our finding of higher prevalence of LTBI among Afghans. Domestic clinicians screening newly arrived SIVH should refer to the CDC’s *Guidelines for the US Domestic Medical Examination for Newly Arrived Refugees* to ensure they are appropriately screening for and treating LTBI, with special attention to adults, who are not routinely tested for *Mycobacterium tuberculosis* infection in the overseas medical exam…’  **Other interpretation of results provided in further detail in the manuscript. |
| 21 | Discuss the generalisability (external validity) of the study results | ‘Given that sites collected and shared data differently, and we did not capture detailed information on method or type of testing for most conditions, our findings only reflect those sites contributing data and not all SIV populations in the US.’ |
| 22 | Give the source of funding and the role of the funders for the present study and, if applicable, for the original study on which the present article is based | ‘CDC collaborated with nine partners as part of a cooperative agreement (*CK12-1205*) to collect domestic screening exam data for analysis.’ |
